# 1618. Assessing and Improving Accurate Diagnosis and Reporting of Congenital Syphilis in a Tertiary Care Children's Hospital

**DOI:** 10.1093/ofid/ofad500.1453

**Published:** 2023-11-27

**Authors:** John Flores, Allison H Bartlett, Allison Nelson, Palak Bhagat, Julia Rosebush, Jessica Ridgway

**Affiliations:** University of Chicago Hospital, Chicago, Illinois; University of Chicago Comer Children's Hospital, Chicago, Illinois; University of Chicago Comer Children's Hospital, Chicago, Illinois; University of Chicago Comer Children's Hospital, Chicago, Illinois; University of Chicago Comer Children's Hospital, Chicago, Illinois; University of Chicago Medicine, Chicago, Illinois

## Abstract

**Background:**

Congenital syphilis (CS) occurs due to transplacental passage of active infection to the fetus during pregnancy. Clinical presentation ranges from asymptomatic to severe permanent disability. Nationally, the incidence and prevalence of syphilis among cisgender females ages 15-44 have increased, with proportional increases in rates of CS. In 2020, 19 cases of CS were reported to the Chicago Department of Public Health (CDPH), a 138% increase from 2019 (8 cases). There are circumstances where infants with a non-reactive rapid plasma reagin (RPR-) may potentially contract syphilis in the context of inadequate maternal treatment per national guidelines. (Figure 1) Currently, only infants with RPR reactivity (RPR+) are reported to CDPH, potentially underestimating the true incidence. Our project sought to identify RPR- infants who received treatment to improve reporting to CDPH, more accurately reflect the prevalence of CS in Chicago, and ultimately guide interventions to reduce and eliminate CS.

**Figure 1:**
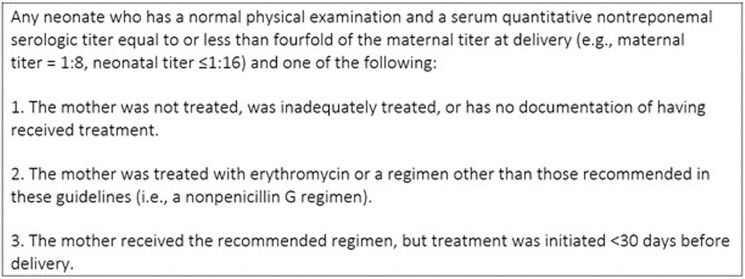
2021 Center for Disease Control (CDC) Possible Congenital Syphilis Definition

**Methods:**

Data was collected via electronic medical record (EMR) review for infants born at Comer Children’s Hospital in Chicago, IL between 2012 and 2022, filtered using ICD-9 code (090) and ICD-10 code (A50.9) for CS. Infants were excluded if diagnosis code was used in error, there was no RPR result, or diagnosis occurred at an outside institution. (Figure 2) Infants were stratified as having CS with RPR+ or RPR-. Retrospectively, each infant was also classified with a diagnosis of less likely CS, possible CS, and proven CS per national guidelines.

**Figure 2:**
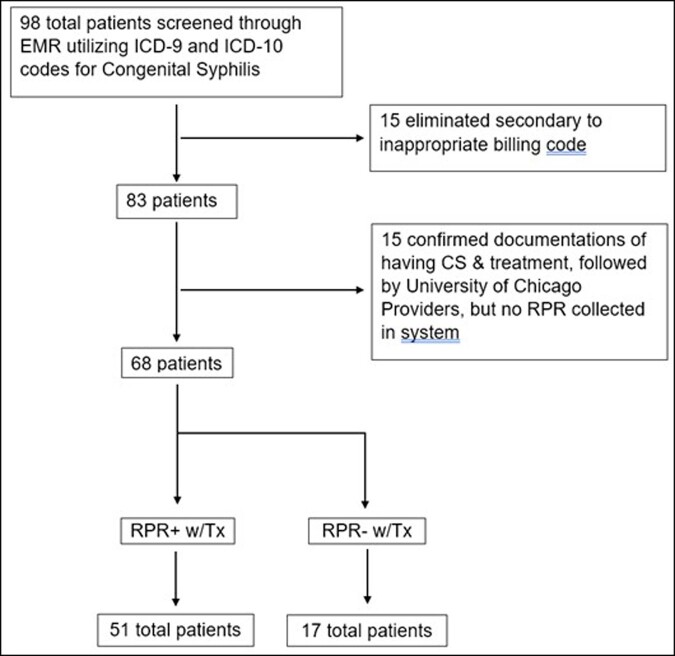
Patient Stratification by ICD-9 and ICD-10 Code for Congenital Syphilis and RPR Reactivity. RPR = Rapid Plasma Reagin

**Results:**

68 infants with CS were identified. 57 infants received IV PCN with diagnoses of proven or possible CS (84%). 51 (75%) patients were RPR+ and 17 (25%) were RPR-. (Figure 3) Of the 17 RPR-, 11 had a diagnosis of possible CS (65%) and received IV PCN while 6 (35%) had less likely CS and received one dose of intramuscular (IM) PCN (Table 1). None of the RPR- infants were reported to CDPH.

**Figure 3:**
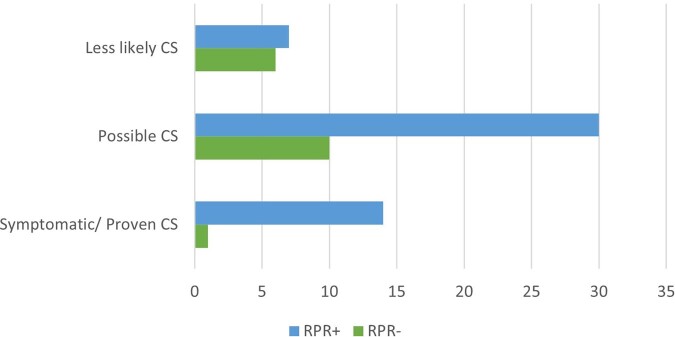
Total Number of Congenital Syphilis Cases Based on CDC Definition. CS = Congenital Syphilis, CDC = Center for Disease Control

**Table 1:**
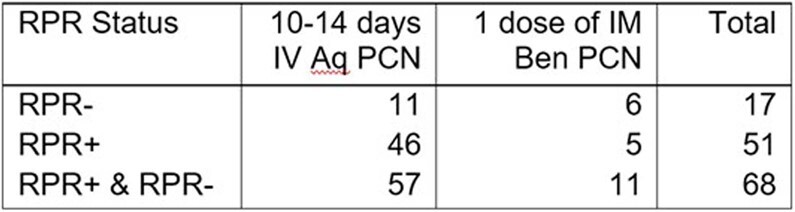
Total Number of Days of Intravenous or Intramuscular Penicillin Administration per RPR reactivity.. Aq PCN = Aqueous Penicillin G, Ben PCN = Benzathine Penicillin G, IM = Intramuscular, IV = Intravenous

**Conclusion:**

In this retrospective analysis, reporting CS based solely on a reactive infant RPR leads to underreporting. As we confront the rising incidence of CS, (Figure 4) it is critical that we have a complete understanding of the epidemiology which may lead to increased support for public health initiatives focused on decreasing and eliminating CS.

**Figure 4:**
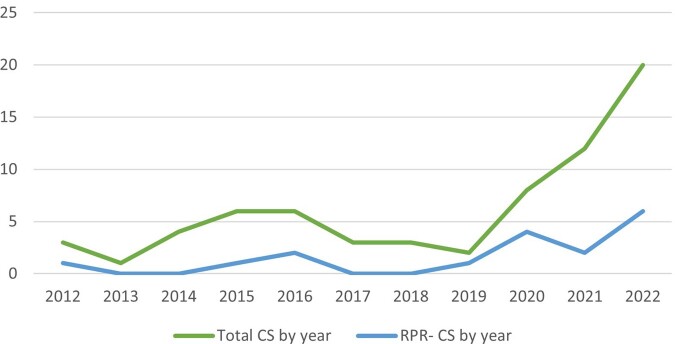
Timeline of Total CS and RPR- CS Cases at Hospital per Year. CS = Congenital Syphilis

**Disclosures:**

**Allison H. Bartlett, MD, MS**, CVS/Caremark: Honoraria **Jessica Ridgway, MD**, Gilead Sciences: Expert Testimony

